# Development of an Implicit Association Test to Assess Healthy and Adaptive Self-Esteem for Lower-Grade Elementary School Students

**DOI:** 10.1192/j.eurpsy.2023.1038

**Published:** 2023-07-19

**Authors:** K. Yamasaki, T. Yokoshima, D. Noguchi, K. Uchida

**Affiliations:** 1 Department of Psychology and Educational Science, Naruto University of Education, Naruto; 2 Center for the Teaching Profession, Shinshu University, Nagano; 3 Department of Early Childhood and Elementary Education, Nakamura Gakuen University, Fukuoka, Japan

## Abstract

**Introduction:**

In recent years, new concepts have been proposed to discriminate between healthy (adaptive) and unhealthy (nonadaptive) self-esteem. Among them, Yamasaki et al. (2017) proposed the concepts of “autonomous” and “heteronomous” self-esteem. As they underscored that autonomous self-esteem needs to be nonconsciously assessed, several nonconscious assessment methods have been developed using implicit association tests (IATs). However, any IAT has not been developed for younger children, even though it is necessary to measure autonomous self-esteem in early childhood because it is largely formed in early developmental stages.

**Objectives:**

The aim was to develop an IAT to measure autonomous and heteronomous self-esteem for 1st-grade elementary school children. In order to standardize it, the validity and reliability were examined.

**Methods:**

Participants were 1st-grade children in a public elementary school in Japan. The final sample included 55 children (35 boys and 20 girls). Their two home-room teachers participated to evaluate the children. The IAT was administered twice, around four weeks apart. The original paper-and-pencil version of the IAT was developed based on the IAT by Yokoshima et al. (2021). In this test, the implicit association between two kinds of stimuli, category (e.g., “myself”) and attribute (face pictograms expressing high and low self-esteem), is measured in terms of accuracy and speed. The evaluation by teachers was conducted using 7-point Likert scales (“not true at all” to “very true”). The evaluation items were “this child proactively does what he or she wants to do alone or with friends for autonomous self-esteem and “this child is competitive and concerned with the consequences of other children” for heteronomous self-esteem.

**Results:**

The test-retest reliability was calculated using correlational analyses of the two measurements. The correlation was significantly positive, *r* = .70, *p* < .01. To examine the validity, the children were divided into high and low groups based on the cut-off scores (over 1 *SD* above the mean and under 1 *SD* below the mean). Statistical analyses showed that the evaluated scores were significantly higher in Group High than in Group Low regarding the characteristic of autonomous self-esteem, *t* (19) = 3.87, *p* < .01, *d* = 1.62, and that they were significantly higher in Group High than in Group Low for the characteristic of heteronomous self-esteem, *t* (19) = 3.14, *p* < .01, *d* = 1.32.

**Conclusions:**

This study showed that the newly developed IAT for lower-grade children includes high reliability and validity. Hereafter, this test can be utilized at schools to assess autonomous and heteronomous self-esteem. As schools are interested in enhancing healthy and adaptive self-esteem, this tool will be an effective assessment method to ascertain how autonomous self-esteem is cultivated.

**Disclosure of Interest:**

None Declared

